# A pilot study on development and feasibility of the ‘MyEducation: CABG application’ for patients undergoing coronary artery bypass graft (CABG) surgery

**DOI:** 10.1186/s12912-022-00814-4

**Published:** 2022-02-04

**Authors:** Z. Noor Hanita, L. A. Khatijah, S. Kamaruzzaman, C. Karuthan, R. A. Raja Mokhtar

**Affiliations:** 1 Department of Nursing Science, Faculty of Medicine, University of Malaya, 50603 Kuala Lumpur, Malaysia; 2 Department of Nursing, School of Medical and Life Sciences, Sunway University, 47500 Selangor, Malaysia; 3 Department of Medicine, Faculty of Medicine, University of Malaya, 50603 Kuala Lumpur, Malaysia; 4 School of Medicine, Faculty of Health and Medical Sciences, Taylor’s University, 47500 Selangor, Malaysia; 5 Department of Surgery, Faculty of Medicine, University of Malaya, 50603 Kuala Lumpur, Malaysia

**Keywords:** Anxiety, Depression, Web-based application, Coronary artery bypass graft surgery

## Abstract

**Background:**

Patients scheduled for coronary artery bypass graft (CABG) surgery tend to have persistent symptoms of anxiety and depression. Course of hospital stay post-CABG procedure has become increasingly shorter over the last few decades. This pilot study was conducted to develop and test feasibility of MyEducation: CABG application as a learning tool to reduce anxiety and depression levels among patients undergoing CABG Surgery.

**Methods:**

This study was quasi-experimental in design. Forty-five patients scheduled for CABG surgery were recruited via consecutive sampling from a Tertiary Referral Centre at Kuala Lumpur, Malaysia. MyEducation:CABG application (Web-based education application) was administered among the intervention group (*N* = 23); while the control group (*N* = 22) underwent standard care. Web-based education application were implemented by nurses at admission and prior to discharge. Patients were assisted in terms of queries and concerns, upon which corresponding information and support was provided. Sociodemographic data were obtained from patients, prior to administration of Hospital Anxiety and Depression Scale which was used to measure levels of anxiety and depression. The educational application was used to obtain satisfaction rating among intervention group. These measures were administered upon admission, on discharge and one-month post-discharge.

**Results:**

Mean anxiety and depression scores among the intervention group were lower compared to the control. This was significant for anxiety upon admission, on discharge and one-month post-discharge (*p* < 0.05). Reduced mean depression scores was only significant at one month post-discharge (p < 0.05). Intervention group were generally satisfied with design, content and usability of the application.

**Conclusions:**

Utilisation of MyEducation: CABG application as an educational platform were associated with reduced anxiety and depression among CABG patients, which probably explains positive user satisfaction levels reported. Hence, the study recommends implementation of this application among larger sample as a way to support patient scheduled for CABG aside, with further possibility of preventing complications.

## Background

Coronary heart disease (CHD) is among the leading cause of morbidity and mortality in both developing and developed countries [1]. Management of heart disease often involve surgical procedures, a form of technological treatment. The recovery process for coronary heart disease (CHD) surgeries, such as coronary artery bypass grafting (CABG) tend to be lengthy and encompasses several technical and clinical challenges. Nonetheless, it remains a frequently performed procedure among cardiothoracic surgeons.

Most patients undergoing CABG surgery for the first time experienced high levels of anxiety and distress for different reasons. But the accompanying decreased quality of life and diminished treatment tolerance could worsen course of disease and recovery. Evidence suggests that the presence of anxiety and depression is to be anticipated among patients scheduled for CABG surgery considering the stressors patients undergoing CABG experienced [2, 3]. Furthermore, presence of anxiety and depression at the preoperative period triggered psychological responses involving the endocrine and autonomic system, influencing postoperative outcomes and length of stay (LOS) [4, 5]. Recent evidence indicated that approximately 30 to 40% of CABG surgery patients experienced a form of psychological depression immediately preceding- and post-surgery [6, 7]. Patients with high levels of anxiety pre-CABG procedure reported more pain, lesser post-surgery symptom-relief and higher readmissions [3, 8, 9].

Nursing professionals play a pivotal role in educating patients regarding pre- and post-operative care as well as recovery process. Cardiac disease nursing knowledge has evolved significantly, placing larger emphasis on self-management as key strategy for health promotion. Yet, guidelines or standards on how to best educate patients undergoing cardiac surgery remain undeveloped [10]. Guo (2014) in a systematic review revealed limited supporting evidence on the positive impact of preoperative education on patients' recovery from cardiac surgery [11]. Further research is required to evaluate the sustained efficacy of cardiac preoperative education intervention and its application among non-Western countries.

Supplemental information is often provided in the form of written pamphlets. But the use of now-indispensable Information and Communication Technology (ICT) could facilitate manual to virtual conversion of said information. Approximately 70% of patients undergoing cardiac surgery accessed the internet for information shortly upon being informed of the procedure, hence making the internet a powerful educational platform [12, 13]. Cognitive behavioral technique based internet programs developed to guide patients through virtual contact with a healthcare professional have emerged a particularly effective option. Therapist-guided online interventions among those without cancer have been as effective as face-to-face interventions for several psychological disorders, including anxiety and depression [14]. As such, a guided web-based self-help application incorporating not only assessments of psychological and physical symptoms, but also information on accessing psychological support could be useful for patients undergoing CABG surgery. This study developed a website to provide preoperative information prior to CABG surgery and was designed to suit mobile technology. Next, it also served as a prospective solution to the time-constraints faced among healthcare providers, which otherwise impacted provision of patient education and support. Besides that, pre-operative instructions supplemented by the website provides an opportunity for patients to address concerns about CABG and self-management. Health care providers, particularly nurses play an important role in educating patients and/or caregiver regarding the procedure and providing information on self-care management in preparation for CABG surgery. In view of the above, development of an educational web-based application for management of CABG may be an effective intervention to reduce progression of psychological symptom among CABG patients. This study investigated effectiveness of a web-based application developed for patients undergoing CABG surgery. The application incorporated preoperative education, early post-operative phase of CABG surgery and subsequently evaluated feasibility of MyEducation: CABG application (web-based application) as a learning tool for pre-CABG surgery patients.

The aim of this study was to evaluate the feasibility of the MyEducation:CABG in reducing anxiety and depression and improving patients satisfaction towards the intervention.

## Methods

### Design and setting

This pilot study utilised a quasi-experimental design to determine the effectiveness of preoperative education by developing a web-based application, the MyEducation: CABG application, and assessed its feasibility in reducing anxiety and depression levels among patients undergoing CABG surgery. The study was conducted in the surgical ward of a Tertiary Referral Centre based in Kuala Lumpur, Malaysia, between April 2017 and September 2017.

### Sample and population

Participants consisted of patients scheduled for CABG surgery. Consecutive sampling was used to recruit 45 patients fulfilling the following inclusion criteria; a) have not had prior elective CABG surgery, b) clinically stable prior to date of recruitment, c) owned or had access to a digital device with internet connection, d) able to provide informed consent and e) able to write, read and speak in both English and Malay languages. The exclusion criteria include patients undergoing emergency surgery, those who have had prior valve surgery or have signs of neurological or cognitive impairments such as vision, hearing or cognitive impairment impeding their ability to use a digital device. This pilot study successfully recruited 45 participants, from which 23 were placed into the intervention group while the remaining 22 remained as control. The reason chose consecutive sampling because of surgery ward is a multidisciplinary ward with five specialties in the one ward: neurology, cardiothoracic, vascular, burns, and general surgery. Randomization of the subjects was challenging as the admission could not be predicted and the subjects that fulfilled the criteria were limited.

### Development process of MyEducation: CABG application and data collection procedure

Nurses have customarily played a pivotal role in patient education. This was a role as vital as the rest of their responsibilities where patient care and well-being was concern; especially among those scheduled for major heart surgeries. The intervention that was used in this study is a Web-based education application, also known as MyEducation:CABG application. MyEducation: CABG application was developed as a preoperative education website to assist patients scheduled for CABG, providing insight on the procedure itself, ways to overcome possible emotional and physical repercussions, as well as preoperative and postoperative self-care management. The application also served as a strategy to extend the scope of nursing services for CABG patients, from hospital to home. This study utilised the ADDIE Instructional Model as a guide to devise instructional materials for the web-based application) [15].

**Fig. 1:**
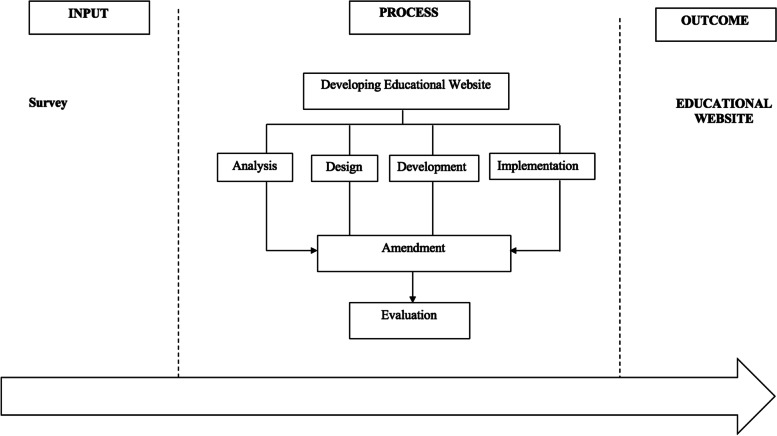
Development process of application through ADDIE model

The entire development process involved five phases; analysis, design, development, implementation and evaluation (ADDIE). The development process is shown in Fig. 1.

#### Analysis phase

This phase involved identifying problems faced by patients through direct interviews by the researcher, followed by a thorough investigation focused on the local setting and a literature review to establish local and international applicability of the intervention. This study found that though cardiac surgery remains routine for cardiothoracic surgeons, it could still evoke anxiety, depression and pain among patients and their families. Furthermore, no formal education program in preparation of the surgery, to address need for information, or to obtain physical and psychological support pre and post procedure was delivered to patients upon admission to the surgical ward. Besides that, the busy ward environment and presence of different surgical cases could pose difficulties for patient in their attempt to manage and retain information, more so when under stress. Subsequently, patients are poorly informed of what to expect over the course of hospitalization.

#### Design phase

This phase focused on conceptual construction of the website, in particular its setting, capacity and usability in fulfilling patient needs. Data collected from the analysis phase was used to design learning content according to patient needs which was then supported through findings obtained from literature review [12–15]. MyEducation:CABG application comprises of six components: Heart; Coronary Artery Disease; CABG Surgery; Recovery Planning Support (i.e. self-management including breathing exercise, medication, turning position, ambulation, eating, and personal hygiene); and My Diary Online (mood and pain level). Figure 1 illustrates the features of MyEducation: CABG application.

#### Development phase

The educational application was developed based on established learning objectives and structure determined from analysis and design phase. Development of the website applications required hardware and selection of correct authoring tools. Authoring tools refers to essential components required to develop software applications, such as audio, video software, graphics, animation, and mobile application development software (e.g. Android developer tools (ADB) or online based software). This website allowing users to enlarge to view the contents and reduce the font size on the screen. This study collaborated with the company, Heartstring, and used Grav®CMS as website development platform considering its quick and easy website building capacity.

#### Implementation phase

This phase involved delivering the MyEducation:CABG application through a smartphone (iPhone and Android). This phase allowed all the materials to be tested to identify if they were functioning well and appropriate for the intended audience. MyEducation:CABG application could be freely accessed by using Chrome 76. Use of the application helped inform of day-to-day event upon patient admission, expectations throughout course of stay, basic information and an overview of normal recovery. It also allowed patients to review information where necessary and enabled participation in personal care process.

#### Evaluation phase

Evaluation was conducted concurrent to the implementation phase, where researchers administered an evaluation survey among 23 CABG patients at the cardiothoracic clinic. Pearson Correlation Coefficient was employed to measure the reliability of two sets of patient’s satisfaction level surveys administered via educational application scores. Cronbach’s α obtained for scale reliability was 0.78. Results revealed Cronbach’s α value of 0.72 (test 1) and 0.76 (test 2) for Patients’ Satisfaction with Educational Application survey, hence demonstrating high level of internal consistency. Further expert review involving the cardiothoracic surgeon, a cardiac rehabilitation clinical specialist, and a cardiothoracic unit nurse was employed to determine the suitability of the educational application.

### Data collection procedure

Every patient who was admitted to the surgery ward (cardiothoracic) and agreed to join in the study was administered the HADS upon admission. The team members who administered the anxiety and depression scale were not aware whether the patient belonged to the experimental or the control group, and neither was the statistician. Patients warded were not offered any written material. All patients received regular pre-operative nursing care, including a tour of the Intensive Care Unit (ICU), shaving, blood test, enema and were informed to be ‘nil by mouth’ after midnight. Ethical consent was obtained from the teaching hospital’s Medical Ethics Committee prior to data collection. Head nurse of each relevant ward were also informed of project details to facilitate cooperation. By using consecutive sampling, it allowed the researcher to start with the control group (April 2017 to June 2017). After completing the control group data collection, a one-week gap was allowed to prevent overlapping or residual samples and the same procedure was applied on the intervention group (July 2017 to September 2017).

The intervention group who received an educational session (MyEducation:CABG application) was kept blinded to the actual purpose of the study. The control group followed the routine hospital protocol (no preoperative education). MyEducation: CABG application was administered via a mobile phone. Patients with smartphones were provided with a link to install the web application. In patients without smartphones, a link was given to their respective caregivers. MyEducation:CABG application session included conversations on what to expect before, during and after CABG procedure; self-management and online diary. Patients were then guided through the installation process beginning with opening the link and uploading the website onto caregiver’s phone. Patients were then taught how to access icons and sound files as well as how to fill-up the diary. All participants were instructed to update the online diary daily. The education session lasted between 15 to 20 min, depending on the patient’s reading speed, interest, and motivation. The session was concluded by reinforcement of information and answering of questions. Education sessions took place preoperative upon admission, as well as during subsequent fourth or fifth postoperative day. After completion of data collection for the intervention group, to ensure equal benefits to both groups, the same web-based education was also given to control group; however, the effects on control group were not measured. Questionnaires were administered to both groups to determine effectivity of MyEducation:CABG application in reducing anxiety and depression levels. Questionnaires were administered on day of hospitalisation, day of discharge and one-month postoperative during follow up visit at the cardiothoracic clinic, respectively. Post-implementation phase was conducted one month postoperative considering the short preoperative period (2 days) and long postoperative period (up to 8 days). Upon discharge, patients were followed-up with one-month post-surgery at the surgical clinic (cardiothoracic), where surgeons assessed them for surgical complications, chest wound and other problems encountered at home. Study by Melike (2019) found that depression and anxiety levels were higher during the 3rd, 7th and 30th postoperative day compared to preoperative period (*P* < 0.001) [16].

### Instruments

The study involved three data collection tools; ‘Personal Information Form’ to obtain patient’s characteristics, ‘Hospital Anxiety and Depression Scale (HADS) [17] to determine levels of anxiety and depression among patients scheduled for CABG surgery and Development of Smartphone Educational Application for patients with CAD to assess user-satisfaction [18].

The Personal Information Form included age, gender, marital status, race, educational level, monthly income, medical comorbidities and waiting list; all of which were collected through individual interviews. While medical comorbidities were obtained via medical records. Folstein’s Mini-Mental State Examination (MMSE) was also administered to screen for cognitive level among patients above 65 years of age to ensure participants were alert, oriented and had no cognitive impairment.

#### Hospital anxiety depression scale (HADS)

The HADS has been repeatedly validated and shown to perform well within the local setting as a screening tool to assess presence and symptom of anxiety and depression [19]. HADS is a succinct, user-friendly, self-reported questionnaire developed by Zigmond and Snaith in 1983 and was used to assess levels of anxiety and depression among patients in nonpsychiatric hospitals. As such, this study administered HADS to prospectively measure anxiety and depression among hospitalised patients awaiting CABG surgery. The questionnaire comprised of 14 items based on two subscales: seven for anxiety (HADS-A) and seven for depression (HADS-D). Each item was rated on a 4-point Likert type scale ranging from 0 (not at all) to 3 (often), with resultant scores possibly ranging from 0 to 21 for each subscale. Scores of 0 to 7 indicated mild anxiety or depression while scores 8 to 14 indicated moderate anxiety or depression. Scores of 15 to 21 indicated severe anxiety or depression [17]. It has been reported that both the English and Bahasa Malaysia versions of HADS had approximately 0.80 sensitivity and specificity [18]. To ensure quality and relevance of the questions towards CABG patients, a panel of expert (i.e. Head of Department of Nursing Science, a clinical specialist cardiothoracic surgeon, a nurse manager of the Cardiothoracic Intensive Care Unit and a cardiac rehabilitation clinical specialist) were engaged to help determine content and face validity of the instrument. Previous studies have assessed validity and reliability of the questionnaire [4, 11]. Nonetheless, this study found an internal consistency reliability value of Cronbach’s alpha 0.74 and 0.67 for anxiety and depression respectively.

#### User satisfaction towards MyEducation:CABG application

At the end of the intervention, a user satisfaction survey was provided to all participants to assess their level of satisfaction towards the application. The instrument was originally devised by developers of the Smartphone Educational Application for patients with CAD [18]. The questionnaire comprised of 12 items evaluated using a 5-point Likert scale ranging from 1 (very dissatisfied) to 5 (very satisfied). The total score ranged from 12 to 60. The higher the mean score indicated that the participants’ perceived satisfaction with the MyEducation:CABG application. Therefore, a total score above 36 is considered as satisfied and less than 35 considered unsatisfied. Feasibility of MyEducation:CABG pilot application was evaluated in terms of content, design, and usability. This instrument was administered among participants upon access to the MyEducation:CABG application. Cronbach’s α reliability value of the scale based on a previous study was 0.95 [12, 20]. Pearson Correlation Coefficient employed in this study to measure the reliability of the two tests found a Cronbach’s alpha value of 0.78. Results obtained revealed a Cronbach’s alpha score of 0.72 (test 1) and 0.76 (test 2) for Patients’ Satisfaction with the Educational Application survey, hence demonstrating high level of internal consistency. This is in addition to expert review by three experts on its educational application.

### Ethical consideration

The research was performed in accordance to the World Medical Association’s Declaration of Helsinki and had been approved by Medical Research Ethics Committee, University Malaya Medical Center, Kuala Lumpur, Malaysia (MEC ID No. is: 201401–0709) where the study was conducted. The study materials (questionnaires and informed consent form) were approved by the ethics committee of the university. Patients received written and oral information relevant to the study procedure and aim as well as potential benefit to consent and participate in the study. We obtained written informed consent from all the participants. The participants were assured about the confidentiality and anonymity of their information.

### Data analysis

Data was analysed via Statistical Package of Social Sciences (SPSS) Version 23. Participants absent during follow-up at cardiothoracic clinic were excluded from analyses. Descriptive statistics were used to display an overview of participants’ socio-demographic with regards to age, gender, marital status, race, educational level, monthly income, medical comorbidities and waiting list. Mean, standard deviation and frequency tables were used to analyse demographic data and questionnaire items. Moreover, descriptive statistics were used to present the patients’ satisfaction using MyEducation:CABG application. T-test, chi-square test of significance and general linear multivariate model (GLM) were used to compare anxiety and depression scores between time of admission, time of discharge and during follow-up visit (one-month after discharge) in both intervention and control group. All reported *p*-values were two-tailed with significance level set at *p* < 0.05.

## Results

### Socio-demographic characteristics of CABG patients

Participants characteristics are shown in Table 1. A total of 45 (100%) participants were involved in this pilot study. Mean age of participants was 57.4 (12.3) and 58.14 (11.78) for the intervention group and control group, respectively. Majority of the participants were male, married, and attained secondary educational level. Statistical analyses revealed no significant difference in terms of sociodemographic distribution between the control group and the intervention group, but significant gender differences and differences in medical comorbidities (*p* < 0.05) existed between these groups.

**Table 1:** Sociodemographic Characteristic of the Respondents (*N* = 45)

Demographic characteristics	Intervention group (*n* = 23)	Control group (*n* = 22)	*p* value
	n (%)	n (%)	
**Age (Mean ± SD)**	57.4 ± 12.3*	58.14 ± 11.78*	0.512 ^b^
**Gender**			
Male	17 (73.9)	19 (86.4)	0.012 ^c^
Female	6 (26.1)	3 (13.6)	
**Marital Status**			
Married	21 (91.3)	18 (81.8)	0.273 ^c^
Divorce/Live alone	2 (8.7)	4 (18.2)	
**Educational Level**			
None	2 (8.7)	5 (22.7)	0.711 ^c^
Secondary	15 (65.2)	14 (63.6)	
Higher	6 (26.1)	3 (13.6)	
**Monthly Income**			
Less<Rm2000	5 (21.7)	4 (18.2)	0.725 ^c^
RM2001 - RM4000	8 (34.8)	10 (45.5)	
Above >RM4001	10 (43.5)	8 (36.4)	
**Medical comorbidities**			
Hypertension	14 (60.9)	12 (54.5)	0.002 ^c^
Diabetes Mellitus	6 (26.1)	6 (27.3)	
Prior Myocardial Infarction	3 (13)	4 (18.2)	
**Waiting list**			
Less than 1 week	7 (30.4)	8 (36.4)	0.320 ^a^
1 week to 1 month	11 (47.8)	12 (54.5)	
More than 1 month	5 (21.7)	2 (9.1)	

^a^mean (SD)

^b^Independent t-test

^c^Pearson Chi-Square test

### Level of anxiety and depression

General linear model (GLM) was used to examine effects of the intervention on anxiety and depression scores between time of admission, discharge, and one-month after discharge. The Multivariate Tests in repeated measures ANOVA indicated that there were significant differences in the anxiety score between the 2 groups before surgery (F = 3.12, *p* = 0.035). Next, a statistically significant difference in anxiety scores were also observed at time of discharge (F = − 2.65, *p* = 0.040) and one-month post-discharge (F = − 0.8, *p* < 0.001), where the intervention group scored lower than the controls. However, for depression scores showed no significant difference between 2 groups before surgery (F = 1.25, *p* = 0.065) and time of discharge (F = -1.92, *p* = 0.071). There were significant differences in the main effect between the 2 groups when one-month after discharge (F = − 4.3, *p* = 0.015). Results from the Hospital Anxiety and Depression Scale preoperative, day of discharge, and one-month post-discharge are as shown in Table 2.

**Table 2:** Effect of MyEducation: CABG application on anxiety and depression level among CABG patients (*N* = 45)

Variables	Level of anxiety and depression		*F*	*p* value ^b^
	Intervention group (*n* = 23)	Control group (n = 22)		
	Mean (SD)	Mean (SD)		
Before surgery (baseline)				
Anxiety	14.21 (4.00)^a^	11.08 (3.37)	3.12	0.035*
Depression	11.25 (2.73)	10.00 (2.23)^a^	1.25	0.065
Day on Discharge				
Anxiety	9.25 (1.90)	11.90 (2.74)	−2.65	0.040*
Depression	7.11 (1.02) ^a^	9.03 (1.25)	−1.92	0.071
One-month post-discharge				
Anxiety	7.30 (4.7)	8.1 (3.21)	−0.8	< 0.001*
Depression	3.9 (3.1)	8.2 (3.25)	−4.3	0.015*

*Significance level *p* < 0.05; ^a^ t-test for independent samples; *SD* Standard Deviation

### User satisfaction towards MyEducation: CABG application

User-satisfaction towards MyEducation:CABG application among patients were strongly influenced by the amount of education received during hospitalization and after discharge. The mean values of user satisfaction according to three domains with the smartphone app are shown in Table 3 depicts patient’s level of satisfaction with the MyEducation:CABG application. Do note that this was only conducted among those within the intervention group (*n* = 23). Majority patients (> 50%) were satisfied with the application’s design, content and usability. The higher item is the information will be useful for me with a mean value is 4.09 (.610).

**Table 3:** Mean Ratings of Patient’s Satisfaction using MyEducation:CABG application (*N* = 23)

Variables	Intervention group (*n* = 23)
	Mean (SD)
**Design of the application n (%)**	23 (100) ^a^
Composition of the application is consistent.	3.84 (.652)
The apps make it easy to learn.	3.77 (.812)
Design, color and font of the screen are very attractive.	3.95(.653)
**Contents n (%)**	17 (73.9) ^a^
Learning objectives are clearly presented.	3.91 (.610)
Content is clear and easy to understand.	3.74 (.621)
Learning contents give us clear information.	3.84 (.531)
The information will be useful for me.	4.09 (.610)
**Usability. n (%)**	15 (65.2) ^a^
It is easy to access the app.	3.28 (1.031)
It is helpful for self-learning.	3.77 (.611)
The app is more interesting than one-on one-education.	3.86 (.560)
The app is an effective strategy for discharge education and can lead to healthy lifestyle.	3.79 (.675)
I want to use the app continuously.	3.86 (.675)

^a^Number of respondents

Patient User’s-satisfaction ratings: 1 = not at all satisfied; 2 = not satisfied; 3 = partially satisfied; 4 = satisfied; 5 = highly satisfied

## Discussion

This pilot study was conducted with the aim of developing a web-based preoperative education application for patients scheduled for CABG surgery. Feasibility of the application was evaluated to determine its utility as a learning tool for patients scheduled for CABG surgery. This included an evaluation of the application’s effectiveness in reducing anxiety and depression symptoms besides identifying user-satisfaction. Initial analyses conducted prior to development of the intervention revealed that anxiety and depression were highly occurring psychological symptoms among CABG patients - a result of which may interfere with scheduling of the surgery and recovery period [4, 9, 21], consistent to that found in other studies [11, 16, 22, 23]. Some patients harboured strong concerns about the surgery and were unable to share their concerns with physicians, inadvertently compromising quality of medical care. Furthermore, misconceptions, insufficient information and previous history of anxiety could lead to fear and anxiety among patients scheduled for surgery, worsening surgical outcome as well as postoperative quality of life [2, 21]^.^ The combined effects of anxiety and depression on physiological mechanisms makes a person in distress psychologically susceptible to cardiac problems [6, 24]. In addition to the fact that cardiac patients have limited access to psychological treatment, it is also important determine whether an easily accessible low-cost intervention, aside from the general health system could be integrated with cardiac patient rehabilitation. Research to date indicate that the number of CABG surgeries affected by anxiety and depression, be it minor, major, and/or dysthymia amount to approximately 30 to 40% [6, 16].

It was imperative for this pilot study to identify pre-operative anxiety and depression among patients undergoing CABG surgery as it enables healthcare professionals to develop effective and appropriate interventions. From this study, it was revealed that more than 50% of the patients had moderate levels of anxiety, with scores ranging from 8 to 14 on the HADS-A, while 51.7% of the patients had mild depression. Consistent with these findings are other published works denoting the negative effect of CABG operation towards psychological status of patients, mostly due to concerns regarding pain and risks of mortality. Furthermore, patients are separated from family, friends, and professional life during preoperative and postoperative periods [14, 25]. Inability to adapt to the aforementioned results in increased anxiety and depression [4]. Accordingly, patients who were highly anxious prior to the CABG procedure experienced more pain, lesser symptom relief following surgery, and frequent readmission. High preoperative anxiety and depression appeared to be predictors of postoperative psychological outcome [11, 23]. This study found depression and CAD to be highly comorbid conditions, with estimation of comorbidity ranging from 14 to 47%. Also, both pre- and postoperative depression were common among patients who underwent cardiac surgery, especially CABG [7, 14]. Significant differences were observed between levels of anxiety and depression within the same group of patients (*p* < 0.001). In our study, patients expressed worries over potential outcomes of the procedure and its complications such as becoming permanently disabled, loss of working ability and fear of death especially young patients. This result is similar to several studies that demonstrated that high anxiety levels among candidates of CABG surgery prior to the procedure, which gradually decreased post-surgery [6, 14, 16]. Another study revealed preoperative anxiety to be higher among CABG patients in comparison to patients hospitalised for other procedures and the general public [2]. These symptoms could result in decreased quality of life as well as physical and mental health among the population.

Results revealed that preoperative levels of anxiety and depression were significantly higher among the intervention group than the control group (*p* < 0.001), but significantly lower at one-month post-procedure (*p* < 0.001). Levels of depression in both groups peaked during time of discharge, which meant that there was no significant difference in depression levels between the two groups during these periods (*p* < 0.001). This finding is consistent with a study by Shahmansouri et al. (2013) investigating prevalence of anxiety and common fears among the CABG surgery patients (*N* = 277) [21] whereby low, moderate and severe anxiety were presented among patients at a prevalence of 19.7, 69.14 and 11.15% respectively. Common fears cited include fear of pain post-surgery, fear of health deterioration, fear of myocardial infarction, and fear of CABG surgery; all of which increased anxiety levels [5]. In addition, Keeping-Burke at al. (2013) found that patients who are followed up by the hospital nurse in the first week after discharge had fewer contacts with their general practitioners [25].

Evidence from this study attested the use of web-based education applications as an effective method to reduce psychological and physical symptoms associated with CABG surgery. The results indicated that a majority of the patients were satisfied with the design, content and usability of the web-based application. The finding of this study was supported with study done by Messerli-Bürgy, Barth & Berger (2012) where in a sample of 59 patients; more than 80% expressed their interest in using a new web-based intervention [26]. Thus, it showed that they were satisfied with the information they obtained from the web-based application. This is further supported by Wang et al. (2017) whose study showed that web-based interventions (i.e. PRECEDE model of health education promotion) were effective in relieving symptoms of depression among older patients with chronic heart failure [15]. However, a study by Norlund et al. (2018) revealed that internet-based Cognitive-Behavioural Therapy (iCBT) treatment did not lower levels of depression or anxiety among population with myocardial infarction [24]. This result however, could have been due to low treatment adherence among the population hence influencing the outcome. A study conducted among 80 patients with depression found that web-based treatments helped improve mental health among depressed cardiac patients with limited access to psychological treatment [26]; suggesting that psychological symptoms associated with hospitalisation could be reduced through multimedia nursing education [27].

The most frequently occurring symptom among our study population were chest incision pain, leg incision pain, shoulder, back or neck pain, as well as anxiety and depression, akin to predominant symptoms of concern among CABG patient reported by previous studies [25, 28–30]. The use of MyEducation:CABG application was effective in reducing anxiety and depression among patients experiencing physical pain (indicated in the form of locality and severity). The application alerted nurse educators to promptly contact the patient in order to assist with symptom management; which proved beneficial to patients who had returned home upon discharge. More than 50% of the respondents were generally satisfied with MyEducation:CABG as an educational application. Moreover, more than 80% of patients expressed interest in using a new web-based intervention, implying satisfaction with information obtained via web-based application. Technology such as smartphones have the capacity to provide hospital services to patients in the comfort of their homes, thus obviating the need for them patients to be in hospital. As hospitals move towards value-based care, it is important that efficiency and cost effectiveness are maintained to ensure preservation of quality, cost, and patient satisfaction towards care provided. There is a growing body of evidence denoting how websites and mobile applications provide opportunities to directly impact delivery of care in a convenient, cost-effective, and scalable manner [31]. It has been widely acknowledged that an effective website is one that is usable and accessible to a wide set of users, including older adults. Messerli-Bürgy, Barth and Berger (2012) in their study involving 59 patients with mean age 67 years old (SD = 8.6) found that 71% patients had access to the internet, and used it to search for information related to health issues on a daily basis [26]. Moreover, more than 80% of patients expressed interest in using a new web-based intervention, implying satisfaction with information obtained via web-based application. Studies by Shahmansouri et al. (2013), and Ventola (2014) found that patients utilising web-based educational programmes revealed improved satisfaction during consultation [20, 31]. These patients however, also reported a longer consultation duration. A web-based education application should adhere to principles of website design for seniors as outlined by the National Institute of Aging (2019) [32]. Thus, the design of web-based education applications should include information fractionated into brief segments, clear language and numbering of each major section. Use of jargon and medical language should be minimised to promote retention and avoid confusion. To allow for ease of interaction with the webpage, a single mouse click should be all that is necessary to access contents, with additional space around clickable targets. The present study presented several limitations. Firstly, the quasi-experimental nature of the study could only offer short-term follow-up among patients. Long-term follow-up with higher number of patients may be beneficial to investigate not only the predictive effect of study characteristics, but also predictors of anxiety and depression associated to CABG surgery. The study also did not assess previous or current use of antidepressant and/or psychiatric services.

## Conclusions

The need for an effective preoperative education programme has become more apparent considering the recent increase in CABG surgeries. MyEducation:CABG application was shown to significantly reduce anxiety and depression levels, although the mechanism leading towards reduction of said symptoms remains unclear. Nevertheless, results obtained encourages use of MyEducation:CABG web-based education application, specifically by nurse educators, towards patients scheduled for CABG surgery. The application offered support in terms of flow and recovery process of CABG procedures and served as an effective tool to reduce anxiety and depression among CABG patients, which in turn could contribute to reduced risk of associated complications. Further research is required to gain deeper understanding on factors mediating such improvements.

## Data Availability

The datasets generated during and/or analysed during the current study are not publicly available due to confidentiality reason but are available from the corresponding author on reasonable request.

## Abbreviations

CABG: Coronary Artery Bypass Graft
CHD: Coronary Heart Disease
MMSE: Folstein’s Mini-Mental State Examination
ICT: Information and Communication Technology
LOS: Length of stay
ADDIE: Analysis, Design, Development, Implementation, Evaluation
GLM: General linear model
